# Validity evidence of two short scales measuring the Big Five personality factors

**DOI:** 10.1186/s41155-018-0111-2

**Published:** 2018-11-27

**Authors:** Jacob Arie Laros, Alexandre José de Souza Peres, Josemberg Moura de Andrade, Maria Fabiana Damásio Passos

**Affiliations:** 1 Instituto de Psicologia, Departamento de Psicologia Social e do Trabalho, Universidade de Brasília, Campus Universitário Darcy Ribeiro, Plano Piloto, Brasília, DF CEP: 70910900 Brazil; 2 Universidade Federal de Mato Grosso do Sul (UFMS), Campus de Paranaíba, Avenida Pedro Pedrossian, n.°725, Bairro Universitário, Paranaíba, MS CEP: 79.500-000 Brazil; 3 Instituto de Psicologia, Departamento de Psicologia Social e do Trabalho, Universidade de Brasília, Campus Universitário Darcy Ribeiro, Plano Piloto, Brasília, DF CEP: 70910900 Brazil; 4 Fundação Oswaldo Cruz, Campus Universitário Darcy Ribeiro - Gleba A, Avenida L3 Norte, SG 10, Brasília, DF CEP 70.904-970 Brazil

**Keywords:** Convergent validity, Big Five personality factors, Reduced scales

## Abstract

The objective of this study was to obtain evidence of the convergent and factor validity of the Reduced Scale of Big Five Personality Factors (ER5FP), with 20 items, and of the Reduced Inventory of Big Five Personality Factors (IGFP-5R), with 32 items. The two Brazilian scales were administered to 554 participants aged 16–69 years (M = 30.6, SD = 8.6). The measurement model of each instrument was tested using confirmatory factor analysis. Both scales showed an adequate adjustment of the measurement model to the data (root mean square error of approximation < .06; standardized root mean square residual < .06) after excluding a number of items. Correlations between the factors of both instruments were estimated. Moderate evidence of convergent validity was found for Extraversion, Neuroticism, and Openness to Experience (raw correlations ranging from .44 and .57, and disattenuated correlations from .60 to .80). For Agreeableness and Conscientiousness, weaker evidence was found (raw correlations of .33 and .29, and disattenuated correlations of .48 and .43, respectively).

## Background

The five-factor model (FFM), also known as Big Five, is currently one of the most influential and investigated models used in the personality research field (De Raad & Mlacic, 2015; McCrae, 2011). In Brazil, although the number of studies published using the FFM as reference is relatively small in comparison to other countries (Silva & Nakano, 2011; Passos & Laros, 2014), it can be stated that the development of instruments based on this model is increasing. Among the psychological instruments approved by the Federal Council of Psychology (Conselho Federal de Psicologia, 2017) and available on the Brazilian market, we can cite the NEO Personality Inventory - Revised (NEO-PI-R (Flores-Mendoza, 2007)), the Factorial Battery of Personality (Bateria Fatorial de Personalidade (BFP) in the original (Nunes, Hutz, & Nunes, 2010)), the Factorial Scale of Neuroticism (Escala Fatorial de Neuroticismo, in the original (Hutz & Nunes, 2001)), the Factorial Extraversion Scale (Escala Fatorial de Extroversão, in the original (Nunes & Hutz, 2007a)), and the Factorial Scale of Socialization (Escala Fatorial de Socialização (Nunes & Hutz, 2007b)). In addition to these, there are a number of instruments under development, either for research purposes or for future commercialization (e.g., Andrade, 2008; Carvalho, Nunes, Primi, & Nunes, 2012; Hauck Filho, Machado, Teixeira, & Bandeira, 2012a,b; Gomes & Golino, 2012; Hutz et al., 1998; Natividade & Hutz, 2015; Passos & Laros, 2015; Primi, Santos, John, & De Fruyt, 2016; Vasconcelos, 2005; Vasconcellos & Hutz, 2008).

Since the instruments for personality assessment tend to be extensive, usually consisting of more than 100 items, one of the research objectives in this area is the development of reduced scales (Carvalho et al., 2012; Hauck Filho et al., 2012a, b; Natividade & Hutz, 2015; Passos & Laros, 2015). The literature shows evidence that a large number of items in an instrument can be a source of measurement error. For example, very long instruments can induce discouragement, fatigue, and inattention in the participants. Furthermore, a large number of items may impede the joint administration of two or more instruments. Therefore, scales with relatively few items can potentially minimize such problems and be useful in different testing settings (e.g., surveys and screenings), provided that they have acceptable reliability indices and satisfactory evidence of validity.

One possible disadvantage of using reduced scales concerns the relatively high amount of measurement errors due to the fact that the number of items of a scale is negatively related to the amount of measurement errors. Another drawback of using reduced scales is related to the possible occurrence of increasing type I and II errors (Credé, Harms, Niehorster, & Gaye-Valentine, 2012). However, the literature already presented evidence that reduced instruments can be a reliable alternative for personality assessment (Donnellan, Oswald, Baird, & Lucas, 2006; Laverdière, Morin, & St-Hilaire, 2013; Rammstedt, 2007). With this motivation, some reduced scales for personality assessment based on the FFM have been proposed in recent years in Brazil.

Andrade (2008), for example, developed the Reduced Inventory of the Big Five Personality Factors (Inventário Reduzido dos Cinco Grandes Fatores de Personalidade (IGFP-5R) in the original), based on the translation and adaptation for Brazil of the Spanish version of the Big Five Inventory (Benet-Martinez & John, 1998). Originally composed of 44 items, the inventory was administered to a sample of 5247 subjects from all Brazilian geographic regions. Confirmatory factor analysis demonstrated the adequacy of a model with 32 items and five correlated factors with reliability coefficients ranging from .66 to .76. By analyzing the items using the item response theory (IRT), the author certified that the instrument had adequate psychometric parameters, even considering that the items did not cover the entire latent trait *continuum*. Among other conclusions, Andrade (2008) pointed out the need to carry out studies investigating the evidence of the convergent validity between the IGFP-5R and other FFM-based instruments, such as NEO-PI-R and NEO-FFI-R.

Hauck Filho et al. (2012a, b) proposed the reduced marker scale (Escala de Marcadores Reduzidos (EMR) in the original), based on adjective markers for personality assessment identified by Hutz et al. (1998). Analyzing data from a sample of 674 university students, a five-factor structure was found. Each factor was composed of five items, with reliability coefficients of factor scores ranging from .61 to .80. In another study with the EMR on a sample of 208 adolescents Hauck Filho et al. (2012a, b), factor analysis also indicated a five-factor structure with each factor containing four items. The reliability coefficients of the factor scores ranged from .55 to .80. Subsequently, Machado, Hauck Filho, Teixeira, and Bandeira (2014) analyzed the responses of 887 university students to the EMR. Factor analysis pointed to a structure with five oblique factors, and IRT analysis indicated a good adjustment of the measurement model, although with a concentration of the items in a restricted range of the latent trait *continuum*, as occurred in the study conducted by Andrade (2008). Finally, Pariz, Haddad, and Machado (2016) gathered evidence of convergent and criterion validity regarding the EMR. The results indicated statistically significant correlations between the EMR and the BFP (Nunes et al., 2010), with the exception of the Socialization (i.e., Agreeableness) and the Openness to Experience factors.

Another example is the study conducted by Carvalho et al. (2012) regarding the translation and adaptation to Brazil of the Ten-Item Personality Inventory (TIPI), developed by Gosling, Rentfrow, and Swann (2003). According to Carvalho et al., TIPI is one of the most cited reduced scales based on the FFM. The results of data analysis of a sample of 404 Brazilian adolescents indicated a structure of three factors, but with low reliability indices ranging from .41 to .63. Among other notes, the authors indicated that before discarding the use of the TIPI in the Brazilian cultural context, it is necessary to gather evidence of validity based on external variables, including other instruments constructed based on the FFM.

Natividade and Hutz (2015) elaborated the reduced scale of personality descriptors (Escala Reduzida de Descritores de Personalidade (Red5) in the original), composed of 20 items. In one study, analyses of a sample of 1889 adults resulted in an orthogonal structure with five principal components with reliability coefficients ranging from .59 to .84. In a second study, with a sample of 512 adults, confirmatory factor analysis corroborated the structure obtained in the first study. There was also evidence of convergent validity between the Red5 and the BFP (Nunes et al., 2010).

Finally, Passos and Laros (2015) proposed a semantic differential scale with 47 items, called Reduced Scale of Big Five Personality Factors (Escala Reduzida de Cinco Grandes Fatores de Personalidade (ER5FP) in the original). Exploratory and confirmatory factor analyses with data from a sample of 365 undergraduate students indicated a five-factor structure with 20 items. The reliability coefficients ranged from .71 to .85. The authors concluded that the scale was adequate as a measure of the five factors, considering the sample analyzed. As a research agenda, the authors highlighted the objective of increasing and diversifying the sample (e.g., the inclusion of participants with other levels of education and from all Brazilian geographic regions) and carrying out convergent and criterion validity studies.

In order to contribute to the development of reduced instruments for the assessment of personality based on the FFM and given continuity to the studies of Passos and Laros (2015) and Andrade (2008), the present study has as overall objective obtaining evidence of the convergent validity between the ER5FP and the IGFP-5R. Moreover, this study aimed to test the factorial validity of the two instruments, analyzing the theoretically expected measurement model for the two scales (i.e., five correlated factors). Finally, this study also had the objective of analyzing the role of covariates in the description of the factors that compose the scales.

The covariates gender, age, and level of education were used to carry out comparisons between groups. The covariant sex was chosen based on studies that indicate that there are differences between men and women regarding personality traits (Schmitt et al., 2017; Weisberg, DeYoung, & Hirsh, 2011). Schmitt, Realo, Voracek, and Allik (2008) concluded in their study with data from 55 nations that in more prosperous and egalitarian societies, the personality traits of men and women tend to be less similar. In general, previous findings indicate that women have higher scores on Extraversion, Agreeableness, and Neuroticism (Weisberg et al. 2011). The effect of age on personality traits has also been investigated in several studies (Donnellan & Lucas, 2008; Rammstedt, 2007; Srivastava, John, Gosling, & Potter, 2003). In a study with a reduced version of the Big Five Inventory, the BFI-10, Rammstedt (2007) concluded that younger participants tended to present higher scores on extraversion, while the older ones had the highest scores on Agreeableness and Conscientiousness. Regarding the effect of level of education, the findings in the studies of Rammstedt (2007) indicated that people with higher levels of education tend to present higher scores on Openness to Experience. In addition to the covariates cited above, we also analyzed variables informing marital status (see Boyce, Wood, & Ferguson, 2016) and whether the participant is a parent (see Prinzie, Stams, Deković, Reijntjes, & Belsky, 2009) in order to verify whether there is any association of these two variables with the personality traits assessed by both instruments.

## Method

### Participants

The sample of this study is composed of 554 subjects (Table 1), mostly female (58.9%) and with a mean age of 30.6 years (SD = 8.61). Regarding the level of education, 1.5% reported having started or finished the primary education level, 29.7% the upper secondary, 51.4% the first stage of tertiary education, and 23.3% graduate school (i.e., *lato* sensu specialization, master or doctoral degrees). The data were collected in the Distrito Federal and in the Metropolitan Region of Salvador, Bahia. An item of the questionnaire asking about the region of origin of the respondents revealed that 47.5% of the participants were originally from the Center-West region, 39.2% from the Northeast, and 13.3% from other geographical regions of the country.

**Table 1:** Sociodemographic information of the participants (*N* = 554)

Variable	Response category	N	%
Sex	Male	226	40.80
	Female	324	58.50
	Not informed	4	.70
Age (years)	< 20	41	7.40
	21–25	122	22.00
	26–30	164	29.60
	31–35	92	16.60
	> 36	134	24.20
	Not informed	1	.20
Marital status	Married	289	52.20
	Single	210	37.90
	Other status	55	9.90
	Not informed		
Level of education	Primary	7	1.20
	Upper secondary	131	23.60
	Tertiary education (first stage)	282	50.40
	Graduate school*	128	23.10
	Not informed	5	.90
Originary geographic region	North	17	3.10
	Northeast	217	39.20
	Center-West	263	47.50
	Southeast	52	9.40
	South	4	.90

*lato sensu specialization, master or doctoral degree

### Instruments

#### Reduced Scale of Big Five Personality Factors (ER5FP)

The ER5FP (Passos & Laros, 2015) is a semantic differential rating scale composed of 20 pairs of bipolar adjectives, with a 7-point Likert type response scale. In the study of Passos and Laros, satisfactory reliability coefficients of the factor scores were found (Extraversion = .85, Conscientiousness = .78, Agreeableness = .81, Neuroticism = .80, and Openness to Experience = .71).

#### Reduced Inventory of the Big Five Personality Factors (IGFP-5R)

The IGFP-5R is an instrument based on the Big Five Inventory (BFI) that was submitted to a validation process for the Brazilian context by Andrade (2008). The IGFP-5R is composed of 32 items, with a 5-point Likert type response scale. The reliability coefficients of the factor scores found in the Andrade study were considered acceptable (Extraversion = .76, Conscientiousness = .66, Agreeableness = 0.74, Neuroticism = 0.75, and Openness to Experience = .68).

### Procedures

#### Data collection

The researchers followed the guidelines of the Resolution 510/2016 of the Brazilian National Health Council (Conselho Nacional de Saúde) regarding scientific research involving human beings. One of the authors recruited the participants and collected the data in one public and two private educational institutions. Prior to the basic instructions, the participants were informed about the research objectives and the confidentiality concerning their identity and signed the Free and Informed Consent Term. The participants individually answered a form, with completion time ranging from 10 to 30 min. The form was composed by the socioeconomic questionnaire, the ER5FP, and the IGFP-5R in this order.

#### Data analysis

The data analysis occurred in four stages. The first stage involved cleaning the database and performing exploratory analyses. Participants who did not respond to one of the instruments or did not report sociodemographic information were withdrawn, as well as those considered outliers based on the Mahalanobis distance. As a result, 9.2% of the cases were excluded, leaving 554 valid cases remaining. Then, the missing data were imputed using the linear trend at point technique of SPSS 18.0. After the exploratory analyses, the assumptions of confirmatory factor analysis (CFA) for the data were checked. First, the assumption of univariate normality was evaluated through the inspection of the asymmetry and kurtosis of the items. An item was considered non-normally distributed if its asymmetry was greater than 1.0 and its kurtosis was superior to 2.0 (Miles & Shevlin, 2001; Osborne, 2002). None of the items of the two personality instruments showed univariate non-normality. Afterwards, the multivariate normality of the items was verified using the multivariate kurtosis coefficient of Mardia (Byrne, 2016).

In the second stage, the CFA of the two instruments was conducted through structural equation modeling with maximum likelihood estimation. The measurement model defined for the two scales aimed the identification of five correlated factors: Extraversion, Conscientiousness, Agreeableness, Neuroticism, and Openness to Experience. Based on the modification indices, changes were made in the initial models of the two scales. Modification indices indicating a substantial impact on the improvement of fit suggested including a correlation between the error terms of several items related to the same factor. Byrne (2016) states that the suggestion of including a correlation between the error terms of two items in the model reveals, in most cases, that the items are very similar in terms of content. In these cases, the author recommends to exclude one of the two items, since the second item does not add information. Thus, the item with the lowest factor loading was excluded of the item pairs for which the modification index suggested correlation between the errors. In the CFA analyses, the recommendations of Byrne (2016), Kline (2016), and Weston, Gore, Chan, and Catalano (2008) were followed.

In the third stage, Guttman’s coefficient lambda 2 (λ2) was calculated to evaluate the internal consistency of the scores of each factor. This coefficient was adopted because it is more appropriate in studies using instruments with a reduced number of items for each latent component (Sijtsma, 2012). In sequence, the raw convergent validity coefficients and the ones corrected for attenuation were calculated. Pearson’s correlation was used to obtain evidence of the convergent validity of the two personality instruments. To correct the raw coefficients for the effect of measurement errors, we used the dual correction formula for attenuation (Osborne, 2013), which is as follows: $$ {r}_{x^{\prime }{y}^{\prime }}=\raisebox{1ex}{${r}_{xy}$}\!\left/ \!\raisebox{-1ex}{$\sqrt{r_{xx}{r}_{yy}}$}\right. $$. In this formula, $$ {r}_{x^{\prime }{y}^{\prime }} $$ is the validity coefficient corrected for the measurement error in both the test and the criterion, *r*_*xy*_ is the observed correlation, *r*_*xx*_ is the reliability of the test, and *r*_*yy*_ is the reliability of the criterion.

The last stage was the analysis of differences between groups on the five-factor scores of each instrument. The groups were selected based on the following covariates: gender (male vs. female), age (less than 30 years old vs. more than 30), being a parent, marital status, and level of education. The statistical package SPPS 18.0 was used in the exploratory and descriptive analyses and for testing the differences between means. The SPSS Amos 18.0 package was employed for the verification of the univariate and multivariate normality, for the design of the factor models and for the CFA.

## Results

### Model fitting

As can be seen in Table 2, the initial ER5FP model has a root mean square error of approximation (RMSEA) value greater than .06 and a standardized root mean square residual (SRMR) value greater than .08, which is not considered a good fit. According to the recommendations of Weston et al. (2008), the minimum criteria for assuring the quality of a model fit are RMSEA ≤ .06 and SRMR ≤ .08. The Comparative Fix Index (CFI) and Goodness of Fit Index (GFI) values of the initial ER5FP model also indicated an unsatisfactory fit since they fell below the .95 criterion. To improve the quality of model fit, alterations in the model were made based on the modification indices suggested by the Amos package. Following the procedure described in the “Method” section, five of the 20 items were excluded from the initial ER5FP measurement model. The fit of the final model with 15 items was satisfactory, with RMSEA = .059, SRMR = .054, and CFI and GFI around .95. Thus, all adjustment indices indicated that the final ER5FP measurement model is appropriate for the data. However, it is important to highlight that the modifications made on the initial model are indicatives that the original structure with 20 items failed to replicate in the sample analyzed in this study.

**Table 2:** Fit indices of the initial and final measurement models of the scales

Scale	Model	Number of items	*χ* ^2^	*χ*^2^/DF	CFI	GFI	RMSEA (IC 90%)	SRMR
ER5FP	Initial	20	710.91	4.43	.83	.88	.079 (.073–.085)	.088
	Final	15	233.57	2.92	.93	.96	.059 (.050–.068)	.054
IGFP-5R	Initial	32	1618.73	3.57	.66	.82	.068 (.065–.072)	.078
	Final	16	169.61	1.80	.95	.96	.038 (.029–.047)	.043

*χ*^2^ chi square, *DF* degrees of freedom, *CFI* Comparative Fix Index, *GFI* Goodness of Fit Index, *RMSEA* root mean square error of approximation, *SRMR* standardized root mean square residual

Similarly, the original structure with 32 items of the IGFP-5R failed to replicate in this study. The original measurement model of the IGFP-5R was also not satisfactory, with RMSEA and SRMR values above the criterion value and with CFI and GFI values below the criterion value (Table 2). Changes were made based on the modification indices to obtain a good fit. Sixteen of the 32 items of the original measurement model were excluded. The fit of the final measurement model was good, with RMSEA = .038, SRMR = .043, CFI = .95, and GFI = .96.

### Reliability coefficients, factor loadings, and correlations between factors

Table 3 displays the factor loadings, the communalities, the correlations between the factors, the item-rest (*r*_*ir*_) correlations, the mean correlation between the items, and the reliability coefficients of the scores on the factors regarding the ER5FP. It is noteworthy that, even with a smaller number of items, the reliability of the factor scores is reasonable, except for the last factor, Openness to Experience, which showed a reliability coefficient of .58. The reliability coefficients of the other factors ranged between .67 and .79, with the greater value referent to the Extraversion factor, which also presented the highest mean correlation among its items (*r* = .56).

**Table 3:** ER5FP: reliability coefficients (Guttman’s *λ*2), factor loadings (FL), communalities (*h*^2^), item-rest correlations (*r*_*ir*_), and correlations between factors (*N* = 554)

Factor 1—Extraversion (*λ*2 = .79)	FL	*h* ^2^	*r*_*ir*_
Item 1. *Extrovertido* (extraverted)/*Tímido* (timid)	.69	.48	.60
Item 5. *Comunicativo* (communicative)/*Calado* (silent)	.87	.76	.69
Item 6. *Sociável* (sociable)/*Reservado* (reserved)	.69	.48	.60
Mean (mean correlation between the items = .56)	.75	.57	.69
Factor 2—Conscientiousness (*λ*2 = .67)	FL	*h* ^2^	*r*_*ir*_
Item 2. *Persistente* (persistent)/*Desistente* (quitting)	.64	.41	.49
Item 10. *Motivado* (motivated)/*Desmotivado* (unmotivated)	.77	.59	.54
Item 12. *Obstinado* (obstinate)/*Inconstante* (fickle)	.51	.26	.41
Mean (mean correlation between the items = .40)	.64	.42	.48
Factor 3—Agreeableness (*λ*2 = .69)	FL	*h* ^2^	*r*_*ir*_
Item 4. *Simpático* (sympathetic)/*Antipático* (unpleasant)	.65	.42	.52
Item 7. *Amoroso* (amorous)/*Indiferente* (indifferent)	.62	.38	.47
Item 14. *Gentil* (kind)/Rude	.70	.49	.53
Mean (mean correlation between the items = .43)	.66	.43	.51
Factor 4—Neuroticism (*λ*2 = .74)	FL	*h* ^2^	*r*_*ir*_
Item 3. *Calmo* (calm)/*Nervoso* (nervous)	.69	.48	.58
Item 13. *Paciente* (patient)/*Impaciente* (impatient)	.77	.59	.58
Item 18. *Tranquilo* (tranquil)/*Ansioso* (anxious)	.63	.40	.54
Mean (mean correlation between the items = .49)	.70	.49	.57
Factor 5—Openness to Experience (*λ*2 = .58)	FL	*h* ^2^	*r*_*ir*_
Item 8. *Criativo* (creative)/*Prosaico* (prosaic)	.57	.32	.42
Item 9. *Entusiasta* (passionate)/*Apático* (apathetic)	.59	.35	.40
Item 15. *Autêntico* (authentic)/*Simulado* (feigned)	.50	.25	.32
Mean (mean correlation between the items = .30)	.55	.31	.38
Correlations between the factors*: F1–F2 = .21 (.40); F1–F3 = .14 (.25); F1–F4 = −.01 (−.02); F1–F5 = .22 (.47); F2–F3 = .29 (.63); F2–F4 = −.14 (−.27); F2–F5 = .33 (.86); F3–F4 = −.13 (−.26); F3–F5 = .26 (.66); F4–F5 = −.04 (−.10)			

*The coefficients corrected for attenuation are between parentheses

Regarding the raw correlations between the factors, the highest positive value was observed between Openness to Experience and Conscientiousness (*r* = .33), while the highest negative values were observed between Neuroticism and Conscientiousness (*r* = −.14) and between Neuroticism and Agreeableness (*r* = −.13). The observed values of the correlations between the five factors have similarities with the results of the study conducted by Herzberg and Brähler (2006) on the evaluation of reduced scales. Analyzing the 16-Adjective Measure, the authors also found negative correlations between Neuroticism and Conscientiousness (*r* = −.09) and positive correlations between Openness and Agreeableness (*r* = .23).

Table 4 displays the same information for the IGFP-5R. The reliability coefficients of the factors are reasonable, ranging from .65 to .72. The correlations between the items of each factor ranged from .34 to .46, with the highest value related to the Neuroticism factor. Concerning the raw correlations between the factors, the highest positive coefficient was observed between Agreeableness and Openness (*r* = .15), while the highest negative coefficient was observed between Neuroticism and Conscientiousness (*r* = −.22). This information is consonant with the results regarding the ER5FP and the findings of Herzberg and Brähler (2006).

**Table 4:** IGFP-5R: reliability coefficients (Guttman’s *λ*2), factor loadings (FL), communalities (*h*^2^), item-rest correlations (*r*_*ir*_), and correlations between factors (*N =* 554)

Factor 1—Extraversion (*λ*2 = .65)	FL	*h* ^2^	*r*_*ir*_
Item 12. *É reservado* (Is reserved)	.50	.25	.41
Item 16. *É*, *às vezes*, *tímido*, *inibido* (Sometimes is timid, inhibited)	.64	.41	.46
Item 42. *Tende a ser quieto*, *calado* (Tend to be quiet, silent)	.70	.49	.50
Mean (mean correlation between the items = .37)	.61	.38	.46
Factor 2—Conscientiousness (*λ*2 = .68)	FL	*h* ^2^	*r*_*ir*_
Item 17. *Pode ser um tanto descuidado* (Can be somewhat careless)	.61	.37	.50
Item 19. *Tende a ser preguiçoso* (Tend to be lazy)	.57	.32	.43
Item 22. *É facilmente distraído* (Is easily distracted)	.57	.32	.44
Item 38. *Tende a ser desorganizado* (Tend to be disorganized)	.61	.37	.47
Mean (mean correlation between the items = .37)	.59	.35	.46
Factor 3—Agreeableness (*λ*2 = .66)	FL	*h* ^2^	*r*_*ir*_
Item 8. *Gosta de cooperar com os outros* (Enjoys to cooperate with others)	.61	.37	.48
Item 15. *É prestativo e ajuda os outros* (Is cordial and helps others)	.76	.58	.55
Item 18. *É amável*, *tem consideração pelos outros* (Is agreeable, cares about the others)	.60	.36	.48
Mean (mean correlation between the items = .43)	.66	.44	.50
Factor 4—Neuroticism (*λ*2 = .72)	FL	*h* ^2^	*r*_*ir*_
Item 10. *É temperamental*, *muda de humor facilmente* (Is temperamental, changes mood easily)	.54	.29	.46
Item 34. *Fica tenso com frequência* (Get tense often)	.70	.49	.55
Item 36. *Fica nervoso facilmente* (Get nervous easily)	.80	.64	.61
Mean (mean correlation between the items = .46)	.68	.47	.54
Factor 5—Openness to Experience (*λ*2 = .67)	FL	*h* ^2^	*r*_*ir*_
Item 9. *É original*, *tem sempre novas ideias* (Is original, always has new ideas)	.69	.48	.51
Item 11. *É inventivo*, *criativo* (Is inventing, creative)	.81	.66	.57
Item 39. *Gosta de refletir*, *brincar com as ideias* (Enjoys thinking, playing with ideas)	.43	.18	.37
Mean (mean correlation between the items = .43)	.64	.44	.48
Correlations between the factors*: F1–F2 = .11 (.25); F1–F3 = .01 (.03**); F1–F4 = .08 (.18); F1–F5 = .05 (.11**); F2–F3 = .12 (.26); F2–F4 = −.22 (−.44); F2–F5 = .04 (.19); F3–F4 = −.11 (−.23); F3–F5 = .15 (.33); F4–F5 = .06 (−.12**)			

*The coefficients corrected for attenuation are between parentheses. **Non-significant correlations, *p* < .05

Table 5 shows the mean, standard deviation, and variance of the scales. For the ER5FP, a comparison was made between the means found in the construction study (Passos & Laros, 2015) and those found in this validity study. For the IGFP-5R, data is presented only for this study. Regarding the distribution of the factor scores, the Agreeableness factor showed the highest mean both for the IGFP-5R (M = 4.48; SD = .61) and the ER5FP (M = 5.71; SD = 1.05). In comparison with the ER5FP development study, the variance of factor scores in this study is lower, especially for Openness and Extraversion (28% and 18% less variance, respectively).

**Table 5:** Statistics of the factor scores of the scales

	ER5FP						IGFP-5R		
	Development study^a^ (*N* = 365)			Convergent validity study (*N* = 554)^b^			Convergent validity study (*N* = 554)		
Factor	M	SD	Var	M	SD	Var	M	SD	Var
Extraversion	4.48	1.71	2.91	4.55	1.54	2.38	2.61	1.01	1.02
Conscientiousness	5.61	1.12	1.26	5.34	1.08	1.17	3.37	0.95	0.90
Agreeableness	5.84	1.07	1.14	5.71	1.05	1.11	4.48	0.61	0.38
Neuroticism	3.95	1.64	2.68	3.81	1.60	2.57	2.74	1.13	1.27
Openness to Experience	5.33	1.13	1.27	5.40	0.96	0.92	3.90	0.75	0.57

*M* mean, *SD* standard deviation, *Var* variance

^a^Passos e Laros (2015)

^b^The factor scores were computed using the same items

### Testing differences between groups

Differences on the five-factor scores of the ER5FP and the IGFP-5R were analyzed between groups of participants based on the following covariates: sex (men vs. women), age (less than 30 years old vs. more than 30), being a parent or not, marital status (single vs. married), and level of education. Concerning the comparisons of means using the ER5FP data, with regard to sex, it was observed that women (M = 3.96) tended to present higher levels of Neuroticism than men (M = 3.59), *p* < .01. When evaluating the covariate age, older people (M1) present higher scores than the younger ones (M0) on Openness (M1 = 5.52, M0 = 5.27, *p* < .05) and Agreeableness (M1 = 5.79, M0 = 5.60, *p* < .05). As for being a parent (M1) or not (M0), the results indicated that the participants who had children tended to be more extraverted (M1 = 4.70, M0 = 4.42, *p* < .05), agreeable (M1 = 5.85, M0 = 5.59, *p* < .01), and open to new experiences (M1 = 5.52, M0 = 5.30, *p* < .01). Finally, although the difference was not large, married participants (M1) had higher scores than the single ones (M0) on Agreeableness (M1 = 5.82, M0 = 5.60, *p* < .01). Using the ER5FP data, no significant differences were found for the level of education, considering the various levels.

The comparisons between groups using the factor scores of the IGFP-5R produced the following results. There were higher rates of Neuroticism among women (M = 2.90) than among men (M = 2.51), *p* < .01. Although the differences were not large, women (M = 4.53) also presented higher scores on the Agreeableness factor than men (M = 4.41), *p* < .05. The results also revealed that males (M = 4.00) tended to be more open to new experiences than females (M = 3.82), *p* < .05. Concerning age differences, older people (M1) tended to show higher indices than the younger ones (M0) on Conscientiousness (M1 = 3.48, M0 = 3.25, *p* < .01) and Agreeableness (M1 = 4.54, M0 = 4.42, *p* < .05), and lower on Neuroticism (M1 = 2.63, M0 = 2.85, *p* < .05). When assessing differences between groups based on the covariate being a parent, it was observed that the participants who had children (M1) presented higher averages on the Agreeableness factor (M1 = 4.56, M0 = 4.42, *p* < .05). Regarding the other covariates, marital status, and level of education, the averages found for the groups did not reveal statistically significant differences.

### Convergent validity evidences

The correlation coefficients corrected for attenuation between the factors of the ER5FP and the factors of the IGFP5-R are shown in the diagonal of Table 6, together with the raw coefficients, which are presented between parentheses. The corrected correlation coefficients can be interpreted as evidence of convergent validity between the instruments. The lower and upper triangles represent evidence of discriminant validity. The corrected coefficients ranged from .43 to .80, while the raw coefficients showed a much lower magnitude, ranging from .29 to .57.

**Table 6:** Raw and corrected for attenuation correlation coefficients between factors of the ER5FP and IGFP-5R scales (*N* = 554)

Factors		IGFP-5R				
		EX	NE	OP	CO	AG
ER5FP	EX	**.80 (.57)**	−.10 (−.07)	−.23 (−.14)	−.20 (−.13)	−.02(−.01)
	NE	−.07 (−.05)	**.60 (.44)**	−.17 (−.11)	−.39 (−.27)	−.28 (−.20)
	OP	.27 (.20)	−.09 (−.06)	**.74 (.46)**	.42 (.28)	.29 (.20)
	CO	.11 (.08)	−.13 (−.09)	.35 (.21)	**.43 (.29)**	.24 (.15)
	AG	.22 (.16)	−.10 (−.07)	.39 (.25)	.31 (.21)	**.48 (.33)**

*EX* Extraversion, *NE* Neuroticism, *OP* Openness to Experience, *CO* Conscientiousness, and *AG* Agreeableness

The raw correlation coefficients are presented between parenthesis and the correlations between the same theoretical factors are in boldface

Considering the validity coefficients corrected for attenuation, Extraversion (*r* = .80), Openness (*r* = .74), and Neuroticism (*r* = .60) showed satisfactory evidences of convergent validity, while Agreeableness (*r* = .48) and Conscientiousness (*r* = .43) were lower than expected. The correlations between different factors also merit attention, such as between Openness to Experience (IGFP-5R) and Conscientiousness (ER5FP), *r* = .42, and between Openness to Experience (ER5FP) and Agreeableness (IGFP-5R), *r* = .39. The magnitude of both corrected and raw coefficients are lower than found in similar studies (cf. Hahn, Gottschling, & Spinath, 2012).

## Discussion and conclusions

After the modifications of the original models (Andrade, 2008; Passos & Laros, 2015), both scales presented adequate psychometric quality in this study, as shown by a good model fit and adequate reliability coefficients (*λ*2). For the ER5FP, with exception of Openness to Experience (.58), the factors showed adequate reliability coefficients ranging from .67 (Conscientiousness) to .79 (Extraversion). For the IGFP-5R, the reliability coefficients varied from .65 (Extraversion) to .72 (Neuroticism). The factors Extraversion and Neuroticism in both instruments presented the highest reliability coefficients and inter-item correlations. This fact may be due to the quality and clarity of the definition of the factors in question, which may have allowed greater coverage of the content of each factor (Frazier, Naugle, & Harggety, 2006). According to cross-cultural studies, Neuroticism and Openness to Experience failed to replicate across different languages (De Raad et al., 2010), which might explain the low reliability of Openness to Experience in the ER5FP.

According to Byrne (2016), after making modifications of the original model, the researcher stops working in a confirmatory mode and starts working in an exploratory fashion. That is the case of this study, once it was necessary to change the models based on modification indices to improve their fit. These changes resulted in the deletion of five items of the original 20 items of the ER5FP and 16 of the original 32 items of the IGFP-5R. Although the five theoretical factors from the FFM model were recovered from the analysis, the loss of items is an indication that the original structure of both instruments failed to replicate in the sample analyzed in this study. Although this data-driven strategy was efficient to improve the fit of the models, it does have limitations. For instance, further studies should contemplate the investigation of the confirmatory validity of the models in independent samples to assess the generalizability of both the original models (Andrade, 2008; Passos & Laros, 2015) and the models identified in this study.

Another related concern is with the loss of items and the content validity of the scales. As other reduced scales, ER5FP and IGFP-5R have a limited coverage of the constructs when compared with long scales. The loss of items as a result of the analyses could potentially reduce this coverage. However, the content of the factors of both ER5FP and IGFP-5R preserved some of the core aspects of the five-factor model—as described in The 100 Revised Synonym Clusters list (Goldberg, 1992; Goldberg & Rosolack, 1994). Concerning the ER5FP, the Extraversion factor contains items related to Gregariousness, Shyness, and Reserve (items 1 and 6) and Expressiveness and Silence (item 5). The Conscientiousness items are associated with Persistence (e.g., item 2) and Decisiveness, Indecisiveness, and Inconsistency (items 10 and 12). The items of the Agreeableness factor are linked to Empathy and Overcriticalness (item 4) and Amiability, Callousness and Rudeness (items 7 and 14). The Neuroticism items are related to Placidity and Fear (items 3 and 18). The Openness items are associated to Creativity and Unimaginativeness (item 8) and Nonconformity (item 15).

In case of the IGFP-5R, the Extraversion items are linked to Reserve (item 12), Shyness and Inhibition (item 16), and Silence (item 42). The items of the Conscientiousness factor are associated to Negligence (item 17), Sloth (item 19), Forgetfulness (Item 22), and Disorganization (item 38). The Agreeableness items are related to Amiability (items 15 and 18) and Cooperation (item 8). The Neuroticism items are linked to Instability (item 10) and Fear (items 34 and 36). Finally, the items on the Openness factor are associated with Creativity (items 9 and 11) and Intellectuality (item 39).

Regarding the convergent validity between the two scales, the correlation coefficients corrected for attenuation showed satisfactory evidence of validity for Extraversion (*r* = .80), Openness (*r* = .74), and Neuroticism (*r* = .60), but not for Agreeableness (*r* = .48) and Conscientiousness (*r* = .43). The magnitude of the raw correlation was even lower. We raise two hypotheses to explain these results. First is the format of the items. While the ER5FP contains bipolar items (e.g., communicative-silent, extraverted-timid, sociable-reserved in Extraversion), the items of the IGFP-5R are unipolar and tend to be emphasize only negative or positive traits in each factor (e.g., reserved, timid, inhibited, quiet, silent in the Extraversion factor). Second is the content of the factors. As one can see in the two previous paragraphs, there are somewhat different emphases in the content of each of the factors. This is also a consequence of the deletion of items in the analyses.

The means of the ER5FP factor scores identified in the development study and in the current study resemble the findings of Donnellan et al. (2006). The data for the Conscientiousness and Openness factors approximate the results presented by the authors regarding the TIPI (Ten-Item Personality Inventory) data, which also have a response scale of 7 points.

Similarities were found comparing differences between groups for the data obtained with the two instruments. On both scales, women presented higher average scores on Neuroticism. Parents obtained higher scores on Agreeableness with both instruments. Concerning the covariate age, the results revealed significant differences for Agreeableness on the two scales, indicating that older people tend to be more agreeable. Regarding marital status, the only difference was on the factor Agreeableness of the ER5FP data, with married participants scoring higher. No differences were found for the level of education. This last result could be related to a homogeneity regarding the educational background in the sample analyzed in this study.

However, exploring the theoretical implications of the observed differences is beyond the scope of this study. Nevertheless, the interested reader can find a number of studies relating the Big Five to a number of covariates such as age (Donnellan & Lucas, 2008; Terraciano, McCrae, Brant, & Costa Jr., 2005), marital status (Boyce et al., 2016), parenthood (Prinzie et al., 2009), and gender (Schmitt et al., 2017; Weisberg et al., 2011).

The sample has other limitations. Because it was carried out basically in the Distrito Federal and in the Metropolitan Region of Salvador, Bahia, the study did not present a representative sample of the entire Brazilian population. In addition, since data collection was performed in educational institutions, there was little variability in the response pattern, which is not the most recommended in studies that seek to evaluate the psychometric quality of instruments. Future studies will benefit if carried out with representative samples, so as to cover the entire national territory.

Investigating convergent validity is an essential step in the development of psychometric instruments and theoretical models. Thus, this study contributes in advancing the research with the FFM, as well as in developing measurement instruments. In addition to investigating the psychometric properties of the instruments, this study may also encourage further research on the relationship between personality and issues related to social context. Finally, the results of this study and those from the works of Passos and Laros (2015) and Andrade (2008) provided preliminary evidence that ER5FP and IGFP-5R are moderately good alternatives for use in future research involving personality assessment within the theoretical framework of the five-factor model.

## Abbreviations

AG: Agreeableness
BFI: Big Five Inventory
BFP: Bateria Fatorial da Personalidade
CFI: Comparative Fix Index
CO: Conscientiousness
DF: Degrees of freedom
EMR: Escala de Marcadores Reduzidos
ER5FP: Reduced Scale of Big Five Personality Factors
EX: Extraversion
FFM: Five-factor model
GFI: Goodness of Fit Index
IGFP-5R: Reduced Inventory of Big Five Personality Factors
NE: Neuroticism
NEO-PI-R: NEO Personality Inventory - Revised
OP: Openness to Experience
Red5: Escala Reduzida de Descritores de Personalidade
RMSEA: Root mean square error of approximation
SRMR: Standardized Root Mean Square Residual
TIPI: Ten-Item Personality Inventory
λ2: Guttman’s coefficient lambda 2
*χ*^2^: Chi square

## Authors’ information

JAL is a professor at the Institute of Psychology of the University of Brasília, Brazil.

AJSP is a professor at the Federal University of Mato Grosso do Sul, Brazil.

JMA is a professor at the Institute of Psychology of the University of Brasília, Brazil.

MFDP is analyst on health management at the Fundação Oswaldo Cruz (Fiocruz), Brazil.

## References

[CR1] Andrade JM (2008). Evidências de validade do inventário dos cinco grandes fatores de personalidade para o Brasil. Tese de Doutorado.

[CR2] Benet-Martinez V, John OP (1998). Los cinco grandes across cultures and ethnic groups: multitrait multimethod analyses of the big five in Spanish and English. Journal of Personality and Social Psychology.

[CR3] Boyce CJ, Wood AM, Ferguson E (2016). For better or for worse: the moderating effects of personality on the marriage-life satisfaction link. Personality and Individual Differences.

[CR4] Byrne BM (2016). Structural equation modeling with AMOS: basic concepts, applications and programming.

[CR5] Carvalho L d F, Nunes MFO, Primi R, Nunes CHS d S (2012). Evidências desfavoráveis para avaliação da personalidade com um instrumento de 10 itens. Paidéia.

[CR6] Conselho Federal de Psicologia. (2017). SATEPSI - Sistema de Avaliação de Testes Psicológicos. ReIRTeved from http://satepsi.cfp.org.br/.

[CR7] Credé M, Harms P, Niehorster S, Gaye-Valentine A (2012). An evaluation of the consequences of using short measures of the Big Five personality traits. Journal of Personality and Social Psychology.

[CR8] De Raad Boele, Barelds Dick P. H., Levert Eveline, Ostendorf Fritz, Mlačić Boris, Blas Lisa Di, Hřebíčková Martina, Szirmák Zsófia, Szarota Piotr, Perugini Marco, Church A. Timothy, Katigbak Marcia S. (2010). Only three factors of personality description are fully replicable across languages: A comparison of 14 trait taxonomies. Journal of Personality and Social Psychology.

[CR9] De Raad B, Mlacic B, Widiger T (2015). The lexical foundation of the big five factor model. The Oxford handbook of the five factor model.

[CR10] Donnellan MB, Lucas RE (2008). Age differences in the Big Five across the life span: evidence from two national samples. Psychology and Aging.

[CR11] Donnellan M. Brent, Oswald Frederick L., Baird Brendan M., Lucas Richard E. (2006). The Mini-IPIP Scales: Tiny-yet-effective measures of the Big Five Factors of Personality. Psychological Assessment.

[CR12] Frazier Thomas W., Naugle Richard I., Haggerty Kathryn A. (2006). Psychometric adequacy and comparability of the short and full forms of the Personality Assessment Inventory. Psychological Assessment.

[CR13] Flores-Mendoza C (2007). Manual do NEO-PI-R e NEO-FFI.

[CR14] Goldberg LR (1992). The development of markers for the Big Five factor structure. Psychological Assessment.

[CR15] Goldberg LR, Rosolack TK, Halverson CF, Kohnstamn GA, Martin RP (1994). The Big Five Factor Structure as an integrative framework: an empirical comparison with Eysenck’s P-E-N Model. The developing structure of temperament and personality from infancy to adulthood.

[CR16] Gomes Cristiano Mauro Assis, Golino Hudson Fernandes (2012). Relações hierárquicas entre os traços amplos do Big Five. Psicologia: Reflexão e Crítica.

[CR17] Gosling Samuel D, Rentfrow Peter J, Swann William B (2003). A very brief measure of the Big-Five personality domains. Journal of Research in Personality.

[CR18] Hahn E, Gottschling J, Spinath FM (2012). Short measurements of personality - validity and reliability of the GSOEP Big Five Inventory (BFI-S). Journal of Research in Personality.

[CR19] Hauck Filho N, Machado WL, Teixeira MAP, Bandeira DR (2012). Evidências de validade de marcadores reduzidos para a avaliação da personalidade no modelo dos cinco grandes fatores. Psicologia: Teoria e Pesquisa.

[CR20] Hauck Filho N, Teixeira MAP, Machado WL, Bandeira DR (2012). Marcadores reduzidos para a avaliação da personalidade em adolescentes. Psico-USF.

[CR21] Herzberg PY, Brähler E (2006). Assessing the Big-Five personality domains via short forms: a cautionary note and a proposal. European Journal of Psychological Assessment.

[CR22] Hutz CS, Nunes CH, Silveira AD, Serra J, Anton M, Wieczorek LS (1998). O desenvolvimento de marcadores para a avaliação da personalidade no modelo dos cinco grandes fatores. Psicologia: Reflexão e Crítica.

[CR23] Hutz CS, Nunes CHS (2001). Escala Fatorial de Neuroticismo.

[CR24] Kline RB (2016). Principles and practices of Structural Equation Modeling.

[CR25] Laverdière Olivier, Morin Alexandre J.S., St-Hilaire France (2013). Factor structure and measurement invariance of a short measure of the Big Five personality traits. Personality and Individual Differences.

[CR26] Machado WL, Hauck Filho N, Teixeira MAP, Bandeira DR (2014). Análise de Teoria de Resposta ao Item de marcadores reduzidos da personalidade. Psico.

[CR27] McCrae RR (2011). Personality theories for the 21st century. Teaching of Psychology.

[CR28] Miles J, Shevlin M (2001). Applying regression & correlation. A guide for students and researchers.

[CR29] Natividade JC, Hutz CS (2015). Escala reduzida de descritores dos cinco grandes fatores de personalidade: prós e contras. Psico.

[CR30] Nunes CHS, Hutz CS (2007). Escala Fatorial de Extroversão.

[CR31] Nunes CHS, Hutz CS (2007). Escala Fatorial de Socialização.

[CR32] Nunes CHS, Hutz CS, Nunes M (2010). Bateria Fatorial de Personalidade (BFP): Manual Técnico.

[CR33] Osborne JW (2002). Notes on the use of data transformations. Practical Assessment, Research & Evaluation.

[CR34] Osborne JW (2013). Best practices in data cleaning: a complete guide to everything you need to do before and after collecting your data.

[CR35] Pariz J, Haddad E, Machado WL (2016). Convergent and criterion-based validity for a brief scale of the five-factor model. Avaliação Psicológica.

[CR36] Passos MF, Laros JA (2014). O modelo dos cinco grandes fatores: revisão de literatura. Peritia – Revista Portuguesa de Psicologia.

[CR37] Passos MFD, Laros JA (2015). Construção de uma escala reduzida de Cinco Grandes Fatores de personalidade. Avaliação Psicológica.

[CR38] Primi R, Santos D, John OP, De Fruyt F (2016). The development of a nationwide inventory assessing social and emotional skills in Brazilian youth. European Journal of Psychological Assessment.

[CR39] Prinzie P, Stams GJ, Deković M, Reijntjes AH, Belsky J (2009). The relations between parents’ Big Five personality factors and parenting: a meta-analytic review. Journal of Personality and Social Psychology.

[CR40] Rammstedt Beatrice (2007). The 10-Item Big Five Inventory. European Journal of Psychological Assessment.

[CR41] Schmitt D, Realo A, Voracek M, Allik J (2008). Why can’t man be more like a woman? Sex differences in big five personality traits across 55 cultures. Journal of Personality and Social Psychology.

[CR42] Schmitt DP, Long AE, McPhearson A, O’Brien K, Remmert B, Shah SH (2017). Personality and gender differences in global perspective. International Journal of Psychology.

[CR43] Sijtsma K (2012). Future of psychometrics: ask what psychometrics can do for psychology. Psychometrika.

[CR44] Silva IB, Nakano TC (2011). Modelo dos cinco grandes fatores da personalidade: análise de pesquisas. Avaliação Psicológica.

[CR45] Srivastava S, John O, Gosling S, Potter J (2003). Development of personality in early and middle adulthood: Set like plaster or persistent change?. Journal of Personality and Social Psychology.

[CR46] Terraciano A, McCrae RR, Brant LJ, Costa PT (2005). Hierarchical linear modeling analyses of the NEO-PI-R scales in the Baltimore longitudinal study of aging. Psychology and Aging.

[CR47] Vasconcelos, T. S. (2005). *O inventário fatorial dos cinco fatores de personalidade no ambiente de trabalho. Tese de Doutorado*. Brasília: Universidade de Brasília.

[CR48] Vasconcellos SJS, Hutz CS (2008). Construção e validação de uma escala de abertura à experiência. Avaliação Psicológica.

[CR49] Weisberg YJ, DeYoung CG, Hirsh JB (2011). Gender differences in personality across the ten aspects of the Big Five. Frontiers in Psychology.

[CR50] Weston Rebecca, Gore Paul A., Chan Fong, Catalano Denise (2008). An introduction to using structural equation models in rehabilitation psychology. Rehabilitation Psychology.

